# The development of the dog heartworm is highly sensitive to sterols which activate the orthologue of the nuclear receptor DAF-12

**DOI:** 10.1038/s41598-020-67466-9

**Published:** 2020-07-08

**Authors:** Thavy Long, Mélanie Alberich, François André, Cécile Menez, Roger K. Prichard, Anne Lespine

**Affiliations:** 1 INTHERES, Université de Toulouse, INRAE, ENVT, 31027 Toulouse Cedex 3, France; 2 Institute of Parasitology, McGill University, Sainte-Anne-De-Bellevue, H9X3V9 QC Canada; 3 Université Paris-Saclay, CEA, CNRS, Institute for Integrative Biology of the Cell (I2BC), 91198 Gif-sur-Yvette, France

**Keywords:** Cell biology, Developmental biology, Microbiology, Molecular biology

## Abstract

Prevention therapy against *Dirofilaria immitis* in companion animals is currently threatened by the emergence of isolates resistant to macrocyclic lactone anthelmintics. Understanding the control over developmental processes in *D. immitis* is important for elucidating new approaches to heartworm control. The nuclear receptor DAF-12 plays a role in the entry and exit of dauer stage in *Caenorhabditis elegans* and in the development of free-living infective third-stage larvae (iL3) of some Clade IV and V parasitic nematodes. We identified a DAF-12 ortholog in the clade III nematode *D. immitis* and found that it exhibited a much higher affinity for dafachronic acids than described with other nematode DAF-12 investigated so far. We also modelled the DimDAF-12 structure and characterized the residues involved with DA binding. Moreover, we showed that cholesterol derivatives impacted the molting process from the iL3 to the fourth-stage larvae*.* Since *D. immitis* is unable to synthesize cholesterol and only completes its development upon host infection, we hypothesize that host environment contributes to its further molting inside the host vertebrate. Our discovery contributes to a better understanding of the developmental checkpoints of *D. immitis* and offers new perspectives for the development of novel therapies against filarial infections.

## Introduction

Nuclear receptors (NRs) are transcription factors involved in key physiological processes such as growth and development, aging and reproduction, as well as metabolism and detoxification of endo- and xenobiotics^1–4^. They typically bind small lipophilic molecules, resulting in their activation, which allows the receptor to recruit co-regulators of expression of specific genes^5^. Because of their central role in critical metabolic processes, including development, NRs are relevant targets for drug discovery^2^. It is estimated that 10–20% of the pharmaceutical market targets these receptors in humans^5^.

NRs are conserved among metazoans including helminths^6^. While humans harbor 49 NRs in their genome, *Caenorhabditis elegans* has 284 NRs^7^ and some are essential for development and reproduction but also survival by enabling this free-living worm to adapt to variable environmental conditions. DAF-12 was described as a NR in *C. elegans*, due to the discovery of its ligands, dafachronic acids (Δ4- and Δ7-DA)^8–10^. In this nematode, DAF-12 acts as a molecular switch in the signaling pathway that regulates dauer formation, or alternative progression to reproductive development and the adult stage, by sensing the environmental quality and bacterial nutrient availability^9,11^. When environmental conditions are favorable, and bacteria are available, *C. elegans* stimulates the production of DA steroid hormones, which activate DAF-12^7,10,12^. CelDAF-12 then induces transcription of development genes that promote growth and reproduction^13–15^. In contrast, in unfavorable conditions, DAs are not synthesized and the DAF-12 apo-receptor interacts with a co-repressor, preventing target gene expression and resulting in arrest of development at dauer diapause larvae, a non-feeding, non-reproducing, stress-resistant, long-lived stage^9,16^. A similar module DA/DAF-12 is functional in other Clade V parasitic nematodes such as the hookworms (*Ancylostoma ceylanicum*, *Ancylostoma caninum*, *Necator americanus*) and the sheep nematode (*Haemonchus contortus*)^17^ as well as in the Clade IV threadworm, *Strongyloides stercoralis*^4,18–22^. Bioinformatic analysis has also revealed that orthologues of DAF-12 exist in the genome of clade I (*Trichinella spiralis*, *Trichuris trichiura*)^23^, and, recently, DA was discovered in *Ascaris suum* and *Toxocara canis* (clade III)^24^. These parasitic nematodes exhibit a direct life cycle in which they develop into a rhabditiform larvae, like *C. elegans*, that can feed on bacteria during free-living development from the first-stage larvae (L1) to the third-stage larvae (L3) stages. The free-living infective L3 (iL3) stage stops feeding and developing (similar to the dauer stage) until it enters a mammalian host. Importantly, DAF-12 also plays a role in the resumption of development to reproductive maturity in the mammalian host. Treatment of *Strongyloides *spp. with DAs, especially with Δ7-DA, inhibits the formation of the iL3 stage or promotes exit from the iL3 stage^20,25^ indicating that targeting the development of iL3 constitutes a strategy to interrupt the life cycle of parasitic nematodes^4,20,26,27^. Although bioinformatics analyses identified orthologues of DAF-12 in the genome of clade III filarial nematodes (*Brugia malayi*, *Loa loa*)^23^, the role of a potential module DA/DAF-12 in those parasites has not been investigated.

*Dirofilaria immitis* is a filarial nematode that affects companion animals, mostly dogs and cats, causing heartworm disease that constitutes a major veterinary concern^28–30^. In 2010, the prevalence of heartworm disease in United States has been estimated to range from 1 to 12% depending on the geographical region^31^. In 2016, the American Heartworm Society (AHS) reported in their triennial survey of veterinary clinics, a 21.7% increase in the number of dogs diagnosed positive for adult heartworm compared with 2013, pointing to the dramatic status of heartworm in US. Since 1987, the treatment of heartworm disease has relied heavily on macrocyclic lactone (ML) drugs. However, the long-term use of ML has led to the development of drug resistance challenging current therapeutic control^32–38^. In the absence of alternative therapeutic strategies such as vaccines, novel drugs to treat heartworm infection are urgently needed. Therefore, studying the mechanisms that govern nematode development is an attractive approach to identifying new therapeutic targets.

The life cycle of filarial nematodes, which belong to Clade III, is markedly different from *C. elegans* and Clade IV and V parasitic nematodes. Filarial nematodes lack any bacteria-feeding rhabditiform free-living stage and the early larval stages are obligatory parasites of an insect intermediate host, such as a mosquito. *D. immitis* initiates its early developing larval stages in a mosquito vector, which transfers, during a blood meal, the iL3 stage into the dog definitive host, providing a new environment suitable for the later developing larval stages and adults. Furthermore, adult females produce live microfilariae, rather than eggs, and the microfilarial stage, which is equivalent to an early L1 stage, remains developmentally quiescent in the mammalian blood stream for up to 2–3 years, until taken up by a mosquito during a blood meal. The signals that trigger these developmental transitions, known as molts because the cuticular hypodermis is renewed, amongst many other changes, are still unknown. It was therefore of interest to identify and characterize the *D. immitis* DAF-12 homolog and investigate its role in the development of the iL3 stage. Here, we built a 3D structure model of *D. immitis* DAF-12 (DimDAF-12) ligand binding domain (LBD) and predicted high affinity binding with small molecules such as DAs and with mammalian cholestenoic acid. Importantly, we demonstrated that these molecules stimulate molting of the *D. immitis* iL3 larvae into L4 probably through their action on DimDAF-12, suggesting a role for DimDAF-12 in *D. immitis* development. This work advances understanding of developmental processes in *D. immitis* and opens perspective for a novel therapeutic strategy to prevent heartworm development at a time when existing heartworm preventives suffer from drug resistance.

## Results

### Identification of a DAF-12 ortholog from *D. immitis*

**Figure 1:**
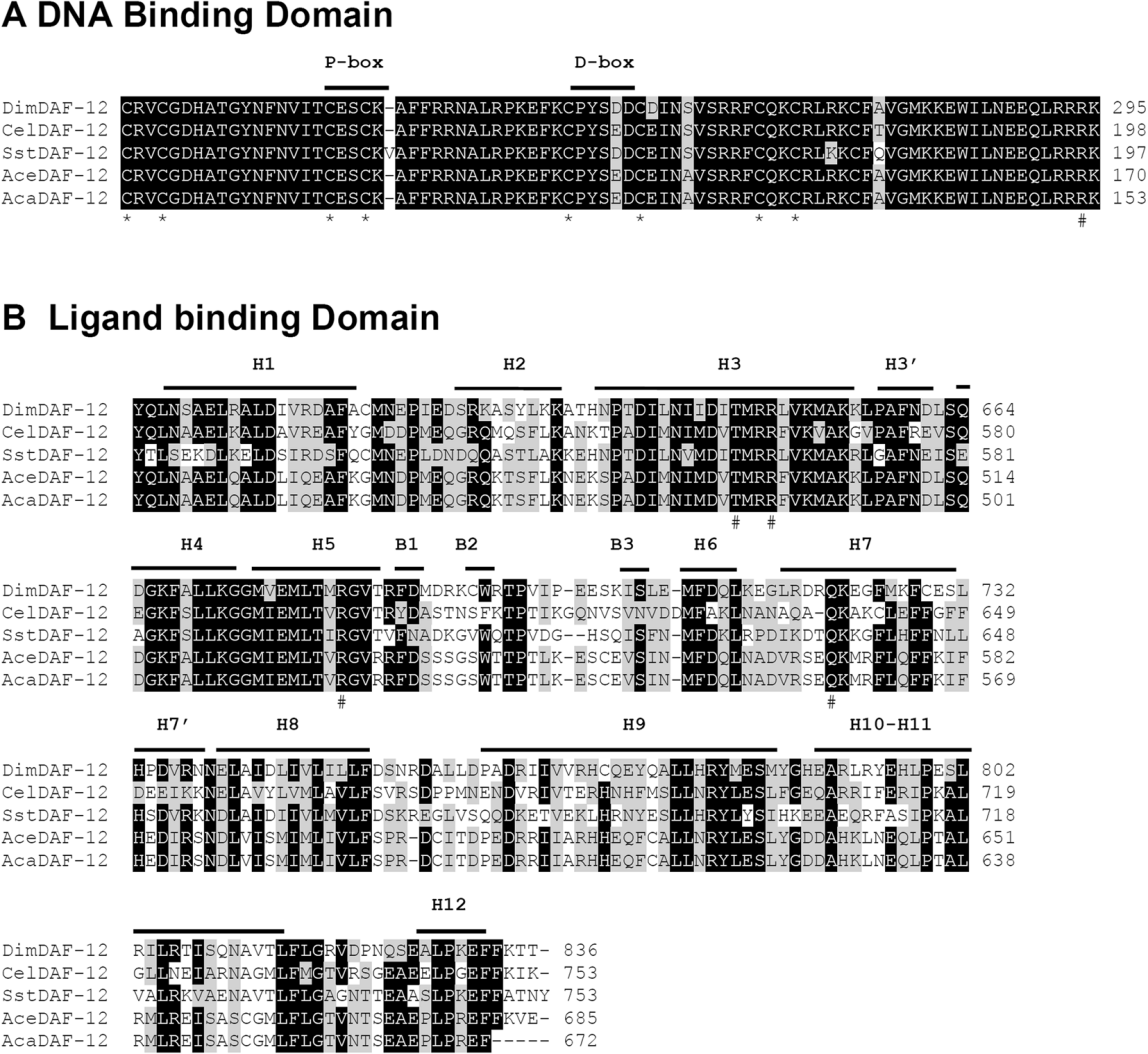
Alignment of DimDAF-12 protein with a subset of known DAF-12 receptors in nematodes. **A** Sequence alignment of the DBD of *D. immitis* (Dim; accession no. MK820661), *C. elegans* (Cel; NP_001024547), *S. stercoralis* (Sst; AAD37372), *A. ceylanicum* (Ace; EPB79655) and *A. caninum* (Aca; RCN38687). The cysteine residues involved in the coordination of zinc atoms are conserved and shown with an asterisk (*) below the sequence. The conserved arginine that is responsible for high affinity DNA binding is shown with a hash mark (#). P-box and D-box are underlined. **B** Sequence alignment of the LBD. The secondary structures delimited above the sequence are based on the crystal structure of SstDAF-12. *H* α-helix, *B* β-sheet. The residues that were previously shown as critical for the binding of dafachronic acids are indicated by hash marks (#) below the sequence. Amino acid residues are indicated on the right. Residues that are identical are highlighted in black and similar sequences in grey. Multiple sequence alignment was performed with BioEdit 7.2 ClustalW Multiple alignment (https://bioedit.software.informer.com/).

Since DAF-12 plays an important role in development of nematodes with free-living stages, we investigated whether DAF-12 is conserved in the obligate parasite, *D. immitis*. We screened the *D. immitis* reference genome nDi.2.2.2 (https://www.nematodes.org/genomes/dirofilaria_immitis/)^39^ with DAF-12 from *C. elegans* and other parasitic nematodes^8,20,21^. BLAST search against the protein database of *D. immitis*, identified a sequence encoding a putative DAF-12. We verified the sequence of the *D. immitis* DAF-12 (DimDAF-12) by amplifying and cloning the cDNA annotated in the genome of *D. immitis* from the start to the stop codons. Analysis of the sequence confirmed that the two domains in DimDAF-12: the DNA binding domain (DBD) and the ligand binding domain (LBD) (Fig. 1)^1,3,40^, are identical to the annotated sequence. However, our sequence analyses revealed two different cDNA sequences which are distinct and shorter than the annotated sequence. Both cDNA lack an indel of 66 bp between amino acids (aa) 562 and 585 respectively, in the short (DimDAF-12a; MK820661) and long sequence (DimDAF-12b; accession no. MN449986). DimDAF-12b differs from DimDAF-12a by the presence of another indel of 24 bp located at the amino acid 158. DimDAF-12a contains an open reading frame (ORF) of 2,487 bp that encodes a protein of 828 aas whereas DimDAF-12b contained an ORF of 2,511 bp that encodes a protein of 836 aas. Since the indel is located upstream of the DBD, it may not impact the activity of DimDAF-12.

DimDAF-12 exhibited overall sequence homologies to other nematode DAF-12 (31–34%) with significant similarities found in the DBD and LBD. DimDAF-12 exhibited 94–98% identity in the highly conserved DBD domain with other nematode DAF-12. The DBD residing at the N-terminus contains two zinc (Zn) finger motifs formed of four conserved cysteines (Fig. 1) that are essential for the binding of NR to specific sequences located in the promoter region of their target genes. The first Zn finger contains the P-box region, which is responsible for high-affinity recognition of the “core half-site” of the HRE. DimDAF-12 DBD shares the exact 13 contiguous conserved residues (TCESCKAFFRRNA) that form the DNA-recognition α helices in CelDAF-12^41^ suggesting that DimDAF-12 might recognize similar DNA sequence as CelDAF-12. The second zinc finger contains the D-box, which provides a dimerization interface. In addition to the two Zn fingers, DimDAF-12 also contains a conserved arginine residue at position 286 that is essential for high-affinity DNA binding. The LBD is located at the C-terminus and appears more divergent between NRs^40^. It contains the ligand binding pocket (LBP) where ligands, co-activators or co-repressors bind to modulate the receptor activity. DimDAF-12 LBD shares only 43% sequence identity to CelDAF-12 but more than 52% with parasitic SstDAF-12, AceDAF-12 and AcaDAF-12. Taken together, the analysis of the sequence showed that DimDAF-12 was a typical NR and structurally related to the DAF-12 NR family.

### Dafachronic acids activate recombinant DimDAF-12

**Figure 2:**
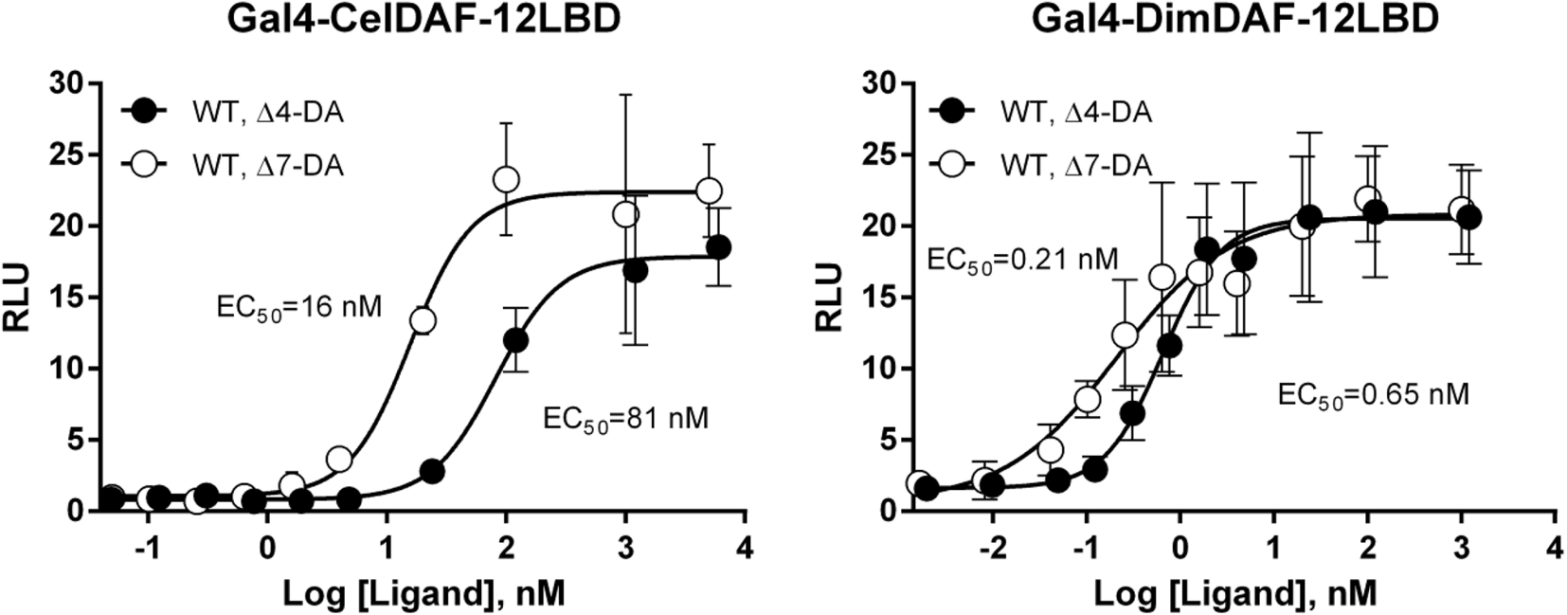
Dafachronic acids activate DimDAF-12 in a dose-dependent manner. NIH3T3 cells were co-transfected either with Gal4-CelDAF-12_LBD (**A**) as control or with the wild-type construct Gal4-DimDAF-12_LBD (**B**) and luciferase gene reporter construct. Recombinant cells were incubated with increasing concentrations of Δ4- or Δ7-DA (respectively up to 5 or 6 µM). The ligand binding and transactivation activity were assessed by measuring the luciferase activity, which was normalized by Renilla luciferase activity for transfection efficiency and expressed as relative light units (RLU). Data represent the average of normalized luciferase activity and the error bars correspond to the standard error of mean from quadruplate assays. The experiments were repeated at least two times in triplicate or quadruplate. The figure shows a representative experiment using Prism 6.0 (Graph Pad Software, Inc.).

Based on the high sequence homologies between DimDAF-12 and the other nematode DAF-12 (Fig. 1), we explored whether DAs, known ligands and activators of CelDAF-12^10,11^, can also activate DimDAF-12. Using a transactivation assay based on mammalian NIH3T3 cells co-transfected with a gene encoding a chimeric protein Gal4-DimDAF-12_LBD and the luciferase gene reporter, we found that both Δ4-DA and Δ7-DA activate the recombinant Gal4-DimDAF-12_LBD protein in a dose-dependent manner (Fig. 2), with EC_50_ of 0.21 and 0.65 nM respectively for Δ7-DA and Δ4-DA whereas the EC50 for activation of CelDAF-12 was 16 and 81 nM respectively.

### Analysis of DimDAF-12 ligand-binding domain structure

To characterize the molecular basis of DA binding mode to DimDAF-12, we constructed a structural model of DimDAF-12_LBD using the coordinates of AceDAF-12 (PDB: 3up0; 1.6 Å) and SstDAF-12 (3gyt; 2.4 Å) LBD crystal structures, for which DimDAF-12 shares respectively 55 and 53% of identity^20,21^. The alignment of DimDAF-12_LBD with 3up0 and 3gyt crystal structure sequences revealed that several helices were conserved such as helix H3, H3′, H4, H5, H6, and H8 (Fig. 1).

Inside the LBD, the LBP contains 30 key residues in CelDAF-12 and SstDAF-12, including mainly hydrophobic residues that can enclose the hydrophobic steroid core of DAs and few polar residues that maintain electrostatic interactions with ligand oxygen atoms (Fig. S1)^20,21,42^. DimDAF-12_LBD shares 22 identical residues with AceDAF-12_LBD in the LBP, while three are replaced by similar amino acids and three present major modifications (F478 to L620, T548 to V690, R574 to G716) (Table S1; Fig. S1). DimDAF-12_LBD shares 23 identical residues in the LBP with SstDAF-12_LBD, four are replaced by similar amino acids and two present major modifications (V603 to R678, A725 to S801) (Fig. S1; Table S1). Remarkably, the DimDAF-12 LBP contains all the conserved key residues involved in the binding of DAs that have been previously identified in the crystal structures (see Supplementary Table S1). In addition, the residue R640 (R564, R498, R565, respectively in Cel/Ace/SstDAF-12) which favors DA binding by forming a lid to entrap the ligand in both Ace and SstDAF-12 structures^20,21^ is also fully conserved.

### In silico prediction of high-affinity binding of DAs on DimDAF-12_LBD model

**Figure 3:**
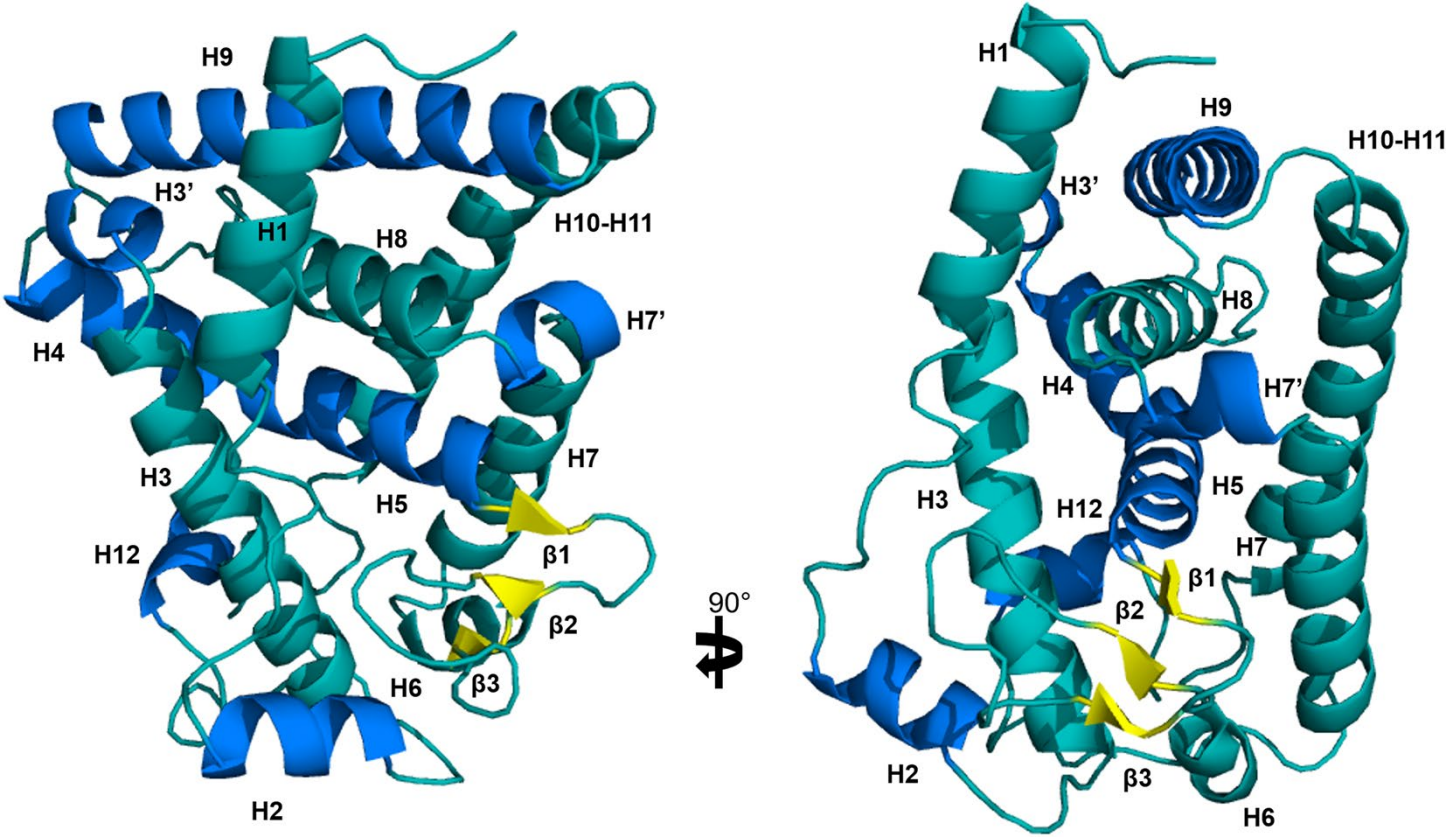
Structure of DimDAF-12 ligand binding domain model. The model of DimDAF-12_LBD (A and B) was constructed using Modeller and the coordinates of the crystal from *Ancylostoma ceylanicum* DAF-12 (pdb:3up0) and *Strongyloides stercoralis* DAF-12 (3gyt). Similar to the other members of the nuclear receptor family, DimDAF-12 exhibits 13 helices and three β-strands, which are packed in a three-layer α-helical sandwich to create a ligand binding pocket, where the DAs were found to bind in the case of AceDAF-12 and SstDAF-12. α-helices were either shown in teal or marine to distinguish them, β-strands were colored in yellow and the loops in teal. All images were generated using PyMol 1.3 (https://pymol.org/2/).

Based on the high sequence similarity in the LBP shared by DimDAF-12 with AceDAF-12 and SstDAF-12, DimDAF-12 might adopt a similar binding mode for DAs. This was investigated by docking experiments. The crystal structures of AceDAF-12 and SstDAF-12 were solved as a complex with respectively, Δ7-DA and Δ4-DA, and we constructed 100 DimDAF-12_LBD apo models using Modeller program and the coordinates of ligand-depleted AceDAF-12 and SstDAF-12 structures. Two models of DimDAF-12_LBD showed the highest accuracy and very similar scores for quality evaluation (Table S2). The model DimDAF-12_LBD_94, which corresponded to the best DOPE score, displayed the maximum number of residues in the allowed regions of the Ramachandran plot and was thus selected for further studies (Table S2). DimDAF-12 adopts a similar LBP as AceDAF-12 and SstDAF-12 (Figs. 1, 3).

**Figure 4:**
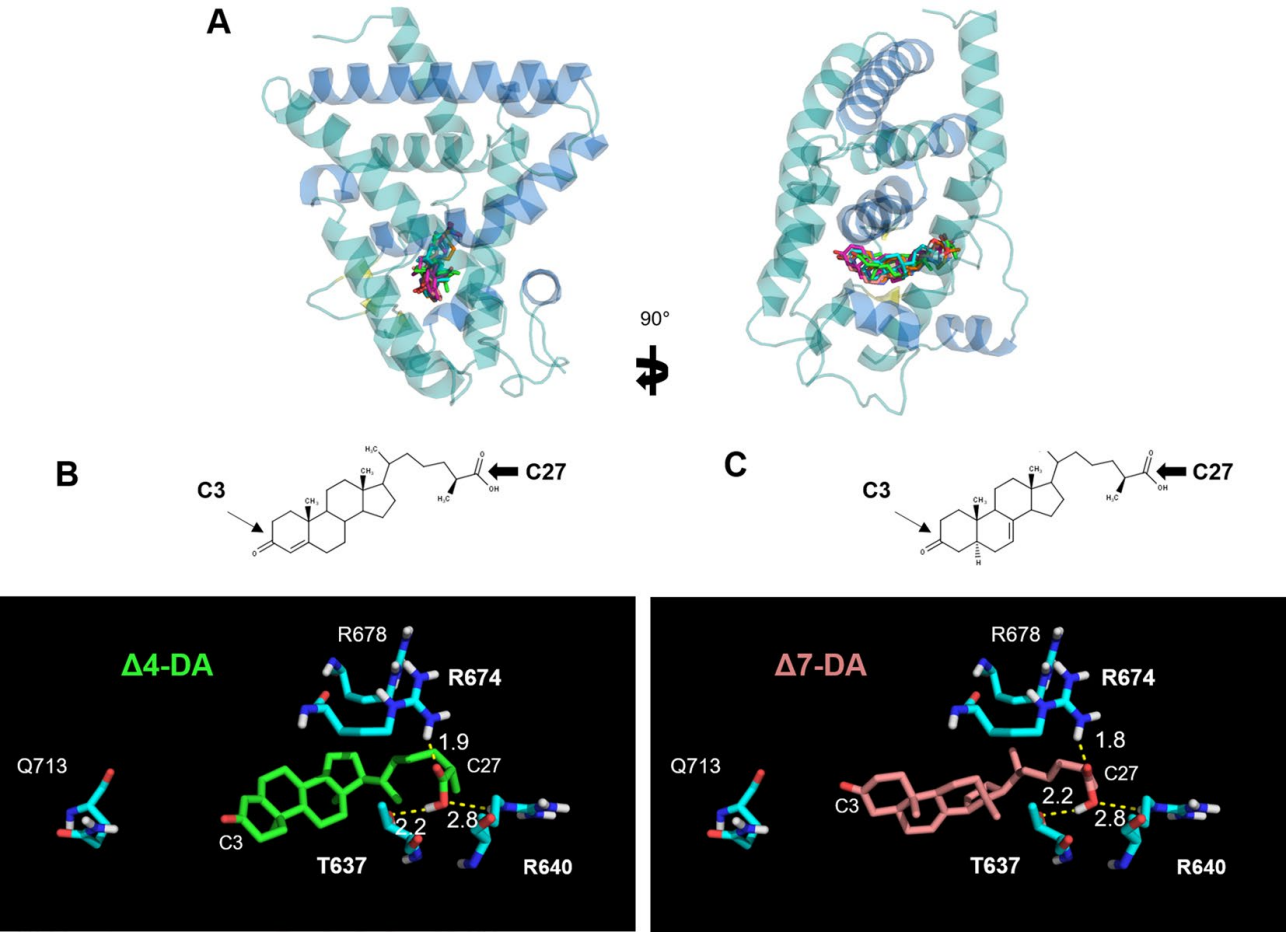
Binding mode of dafachronic acids on model of DimDAF-12 ligand binding domain. **A** In silico docking of dafachronic acids (DAs) into the model of DimDAF-12 LBD was performed using Autodock. Δ4-DA and Δ7-DA bind inside the ligand binding pocket of DimDAF-12. The structures of Δ4-DA (**B**) and Δ7-DA (**C**) differ in the position of an unsaturated double bond respectively at C4 or C7 of the steroid nucleus. H-bonds are illustrated by yellow dashed lines with bond lengths noted in Å. All images were generated using PyMol 1.3 (https://pymol.org/2/).

We evaluated in silico the binding mode of different conformers of Δ4- and Δ7-DA to the model DimDAF-12_LBD_94 (Fig. 4). For all the docking calculations, all the conformers of DAs bound to the LBP with similar binding points and orientations. Δ4-DA and Δ7-DA both possess polar groups at their extremities that are determining for ligand recognition (Fig. 4B): oxygen atoms from the C3 ketone (arrow) and the C27 carboxyl groups (arrowhead) can form H-bonds and/or salt bridges with polar and charged side chains of various residues.

As a result of the docking calculations, the best docking poses in the predicted complex DimDAF-12_LBD/DA involved a slightly different network of interactions compared to the crystal structures of the templates. For all conformers of DAs, the best solution of lowest energy cluster (binding energy score: − 13.3 and − 13.5 kcal/mol for Δ4-DA and Δ7-DA, respectively) (Table S3) revealed two salt bridges established between the ligand carboxylate group and two arginine sidechains (R674 and R640), for both Δ4- and Δ7-DA, whereas only one was detected in the crystal structures, involving the arginine residue equivalent to R674. However, the H-bond interaction involving DAs carboxylate group and threonine residues in the templates (T495 and T562 in Ace and SstDAF-12 respectively) was also found in our best scored conformations with the conserved T637 residue. By contrast, on the opposite moiety of the ligand molecule, our models did not predict any interaction between the C3 ketone group of Δ4- or Δ7-DA and surrounding side chains of DimDAF-12_LBD.

### Several residues confer high-affinity binding of DAs to DimDAF-12_LBD

**Figure 5:**
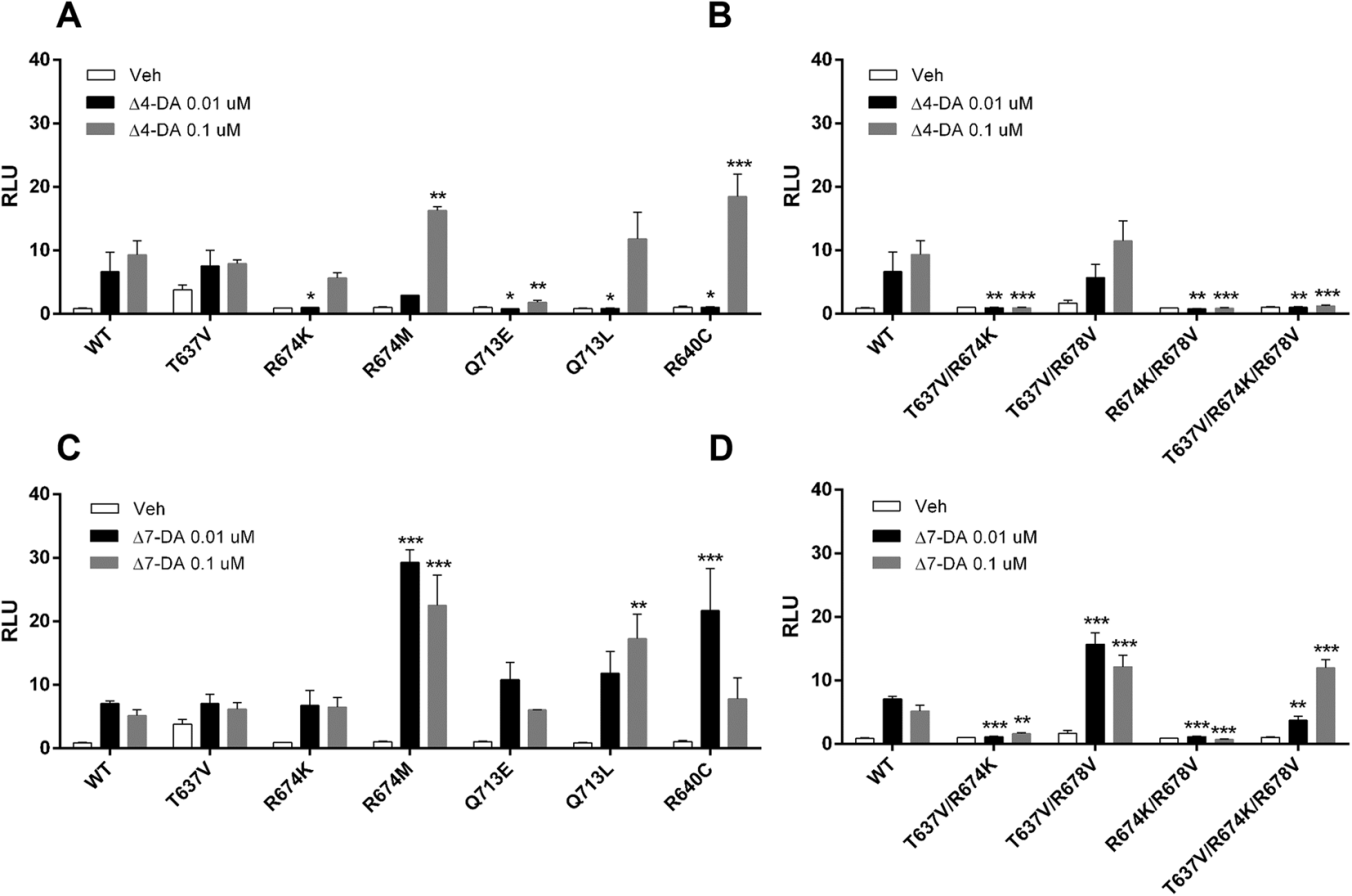
Mutation of residues in DimDAF-12 ligand binding pocket abolishes the activity of the receptor. NIH3T3 cells were co-transfected with wild type Gal4-DimDAF-12_LBD or the corresponding mutants and luciferase gene reporter construct and then tested with 0.01 or 0.1 uM of Δ4- (**A** single mutant, **B** double mutants) or Δ7- (**C** single mutant, **D** double mutants) dafachronic acids (DAs). The ligand binding and transactivation activity were assessed by measuring the luciferase activity, normalized by Renilla luciferase activity for transfection efficiency and expressed as relative light units (RLU). Data represent the average of normalized luciferase activity and the error bars correspond to the standard error of the mean from a triplicate assay. The figure shows a representative experiment performed in triplicate. The significance of the effects of mutation on DimDAF-12 LBD activity was analyzed by two-way ANOVA with multiple comparisons without correction using Prism 6.0 (Graph Pad Software, Inc.) (***p < 0.0005; **p < 0.005; *p < 0.05) versus wild-type.

To confirm the mode of interaction of DAs with DimDAF-12 as revealed by the model, we disrupted the putative H-bonds and ionic interactions by mutating the polar residues T637 and R674 predicted to be essential for binding Δ4-DA and Δ7-DA. We then tested variable mutants with two concentrations of DAs (0.01–0.1 µM corresponding to the concentration where the receptor is completely saturated) in cell-based transactivation assays. First, we found that the single mutation T637V (residue T637 mutated to a valine) had no consequence on the activity of DAF-12 with both DAs (Fig. 5A, C). However, the mutations R674K and R674M had distinct effects on DimDAF-12 activity when tested with either Δ4- or Δ7-DA. While the mutant R674K significantly decreased the Δ4-DA-dependent activity of DimDAF-12 at 0.01 µM, it did not affect the Δ7-DA-dependent activity (Fig. 5A, C).

However, we found that the double mutant T637V/R674K was not active with any of DAs (Fig. 5B, D). In contrast, the activity of the double mutant T637V/R678V was conserved with Δ4-DA and even significantly increased with Δ7-DA (Fig. 5B, D). However, when both residues R674 and R678 were mutated respectively into a lysine and a valine, DimDAF-12 significantly lost DA-dependent activity (Fig. 5B, D) indicating that the H-bond formed by T637 is not crucial for the activity of DimDAF-12, and also confirming that the salt bridge formed with R640 can be disturbed by a repulsive effect of lysine group. This is supported by the absence of activity of R674K/R678V where T637 is intact but apparently not sufficient to activate DimDAF-12 (Fig. 5B, D). The triple mutant T637V/R674K/R678V completely abolished the activation of DimDAF-12 by Δ4-DA, whereas the activity of this triple mutant was significantly increased with Δ7-DA (Fig. 5).

Overall, the results revealed that DimDAF-12 responded differently to mutations compared with SstDAF-12 and other DAF-12s so far examined. To validate the model and confirm that the residue Q713 was not involved in H-bond formation with the C3 ketone of Δ4-DA or Δ7-DA, we also mutated the Q713 to glutamate (Q713E) or leucine (Q713L) and demonstrated that these mutations compromised the activity with Δ4-DA but not with Δ7-DA (Fig. 5A, C), with Q713L increasing the Δ7-DA dependent activity (Fig. 5C). This suggests the absence of a stabilizing H-bond interaction with Q713, and supports the docking computations.

Finally, we mutated the residue R640 to a cysteine residue, which is also conserved among DAF-12 orthologues and has been described as essential for the activity of CelDAF-12 and other parasitic DAF-12^20,21^. DimDAF-12_R640C had differential effects on DimDAF-12 activity: a significant decrease and increase respectively with 0.01 and 0.1 µM of Δ4-DA; and a significant increase with 0.01 µM of Δ7-DA suggesting that the residue R640 would be also important to maintain the activity, as shown by the salt bridge present in our best-scored docked conformations.

### Dafachronic acids accelerate the molting process of *D. immitis* infective third-stage larvae (iL3) into L4

**Figure 6:**
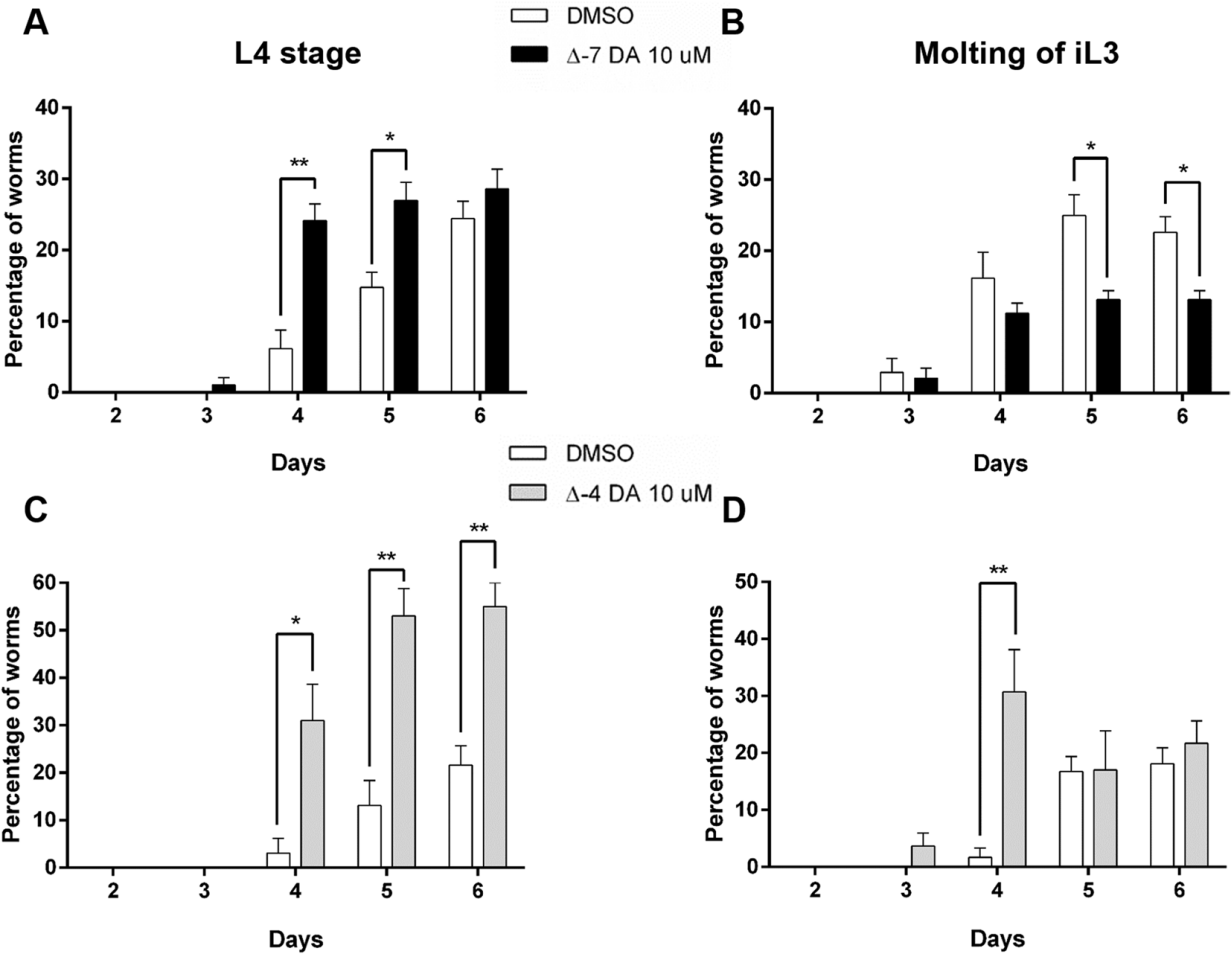
Dafachronic acid accelerates the complete molting of infective third stage *D. immitis* larvae to L4. The iL3 stage larvae were collected from mosquitos and treated with Δ7- (Δ7-DA, **A** and **B**) or Δ4-dafachronic acid (Δ4-DA, **C** and **D**) at 10 µM or DMSO. The developmental changes were observed daily under a microscope. The iL3 that completed the molting from iL3 to L4 (left panel **A** and **C**) were scored as well as the iL3 that were still undergoing molting (right panel **B** and **D**). The number of free cuticles (left panel) found in the wells are directly related to the amount of larvae that had completed the molt and were L4. The figure represents the mean of two independent experiments performed in quadruplate from different pools of iL3s using Prism 6.0 (Graph Pad Software, Inc.). The statistical significance was evaluated with an unpaired t test (**p < 0.005; *p < 0.05).

To evaluate the effects of DAs in vitro on *D. immitis* iL3, we exposed the iL3 to 10 µM of either Δ7-DA or Δ4-DA, one day after recovery from mosquitoes and recorded under a microscope any developmental changes (relative to the molting process). The worms grew in size and stayed alive during the experiment and we did not see any killing effect of DAs. To assess whether DAs impacts the molting of iL3, we observed (1) the shedding of the iL3 cuticle, which corresponded to L3 larvae that had started to molt, and (2) the presence of free cuticles in the medium which was a sign that the molting of iL3 to L4 was completed (Fig. 6A). For Δ7-DA, the molting process started around day 3–4 in culture. Interestingly, we found that the number of free cuticles were significantly higher in Δ7-DA-treated worms than in the DMSO control, by day 4 and 5, suggesting that there were more L4 in Δ7-DA-treated conditions compared to DMSO-treated. This was confirmed by the low percentage of iL3 which had initiated but not completed the molt at day 5 or 6 in the medium containing Δ7-DA, compared to DMSO control demonstrating that Δ7-DA induced a rapid development of iL3 into L4. In the same way, we observed that Δ4-DA accelerates the molting process of iL3 with a significant higher percentage of iL3 that initiated the molting at day 4 and completed the molting at day 5, compared to DMSO (Fig. 6C, D).

### Cholestenoic acid, a mammalian cholesterol metabolite, accelerates the development of iL3 probably through DimDAF-12

**Figure 7:**
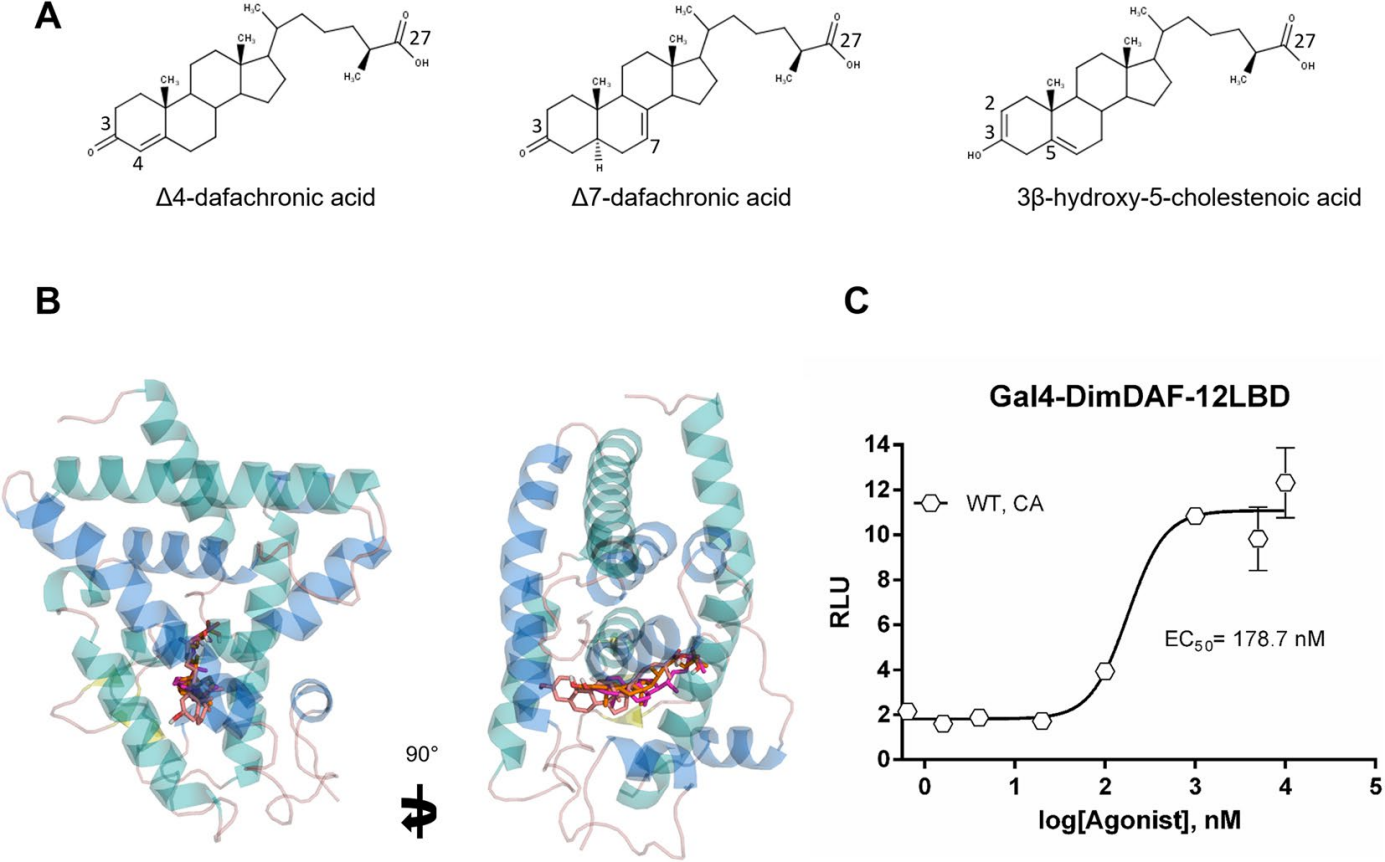
Cholestenoic acid binds and activates DimDAF-12. **A** Chemical structures of Δ4-dafachronic acid (Δ4-DA), Δ7-dafachronic acid (Δ7-DA) and 3β-hydroxy-5-cholestenoic acid (CA). **B** Predicted structures of the complexes of DimDAF-12 formed with Δ4-DA (green), Δ7-DA (pink) and with CA (magenta). In silico docking showed that the ligand binding pocket of DimDAF-12 accommodates CA. All images were generated using PyMol. **C** CA activates DimDAF-12 in a dose-dependent manner. NIH3T3 cells were co-transfected with Gal4-DimDAF-12_LBD and luciferase gene reporter construct. Recombinant cells were incubated with increasing concentrations of CA (up to 10 µM). The ligand binding and transactivation activity were assessed by measuring the luciferase activity, which was normalized by Renilla luciferase activity for transfection efficiency and expressed as relative light units (RLU). Data represent the average of normalized luciferase activity and the error bars correspond to the standard error of mean from a triplicate assay. The experiments were repeated at least two times in triplicate. The figure shows a representative experiment performed in triplicate using Prism 6.0 (Graph Pad Software, Inc.).

Since the recovery of iL3 development and molting of parasitic nematodes appear to be triggered by host environment, we evaluated whether mammalian cholesterol metabolites such as cholestenoic acid (CA), can display in silico binding affinity for the model of DimDAF-12. Cholestenoic acid is an endogenous cholesterol metabolite found in mammalian blood with a core structure similar to DAs that differs by the presence of two double bonds respectively at C2 and C5 of the steroid nucleus and a C3 hydroxyl group instead of a C3 ketone (Fig. 7A). In our structural model of DimDAF-12, we found that CA bound in the LBP with the same orientation and with a similar high binding affinity as DAs (scoring energy − 13 kcal/mol vs. − 13.5 with DAs) (Fig. 7B; Table S4). Consistently, the cell-based transactivation assay revealed that CA activates DimDAF-12 but at a lower potency (EC_50_ = 178 nM) than DAs.

**Figure 8:**
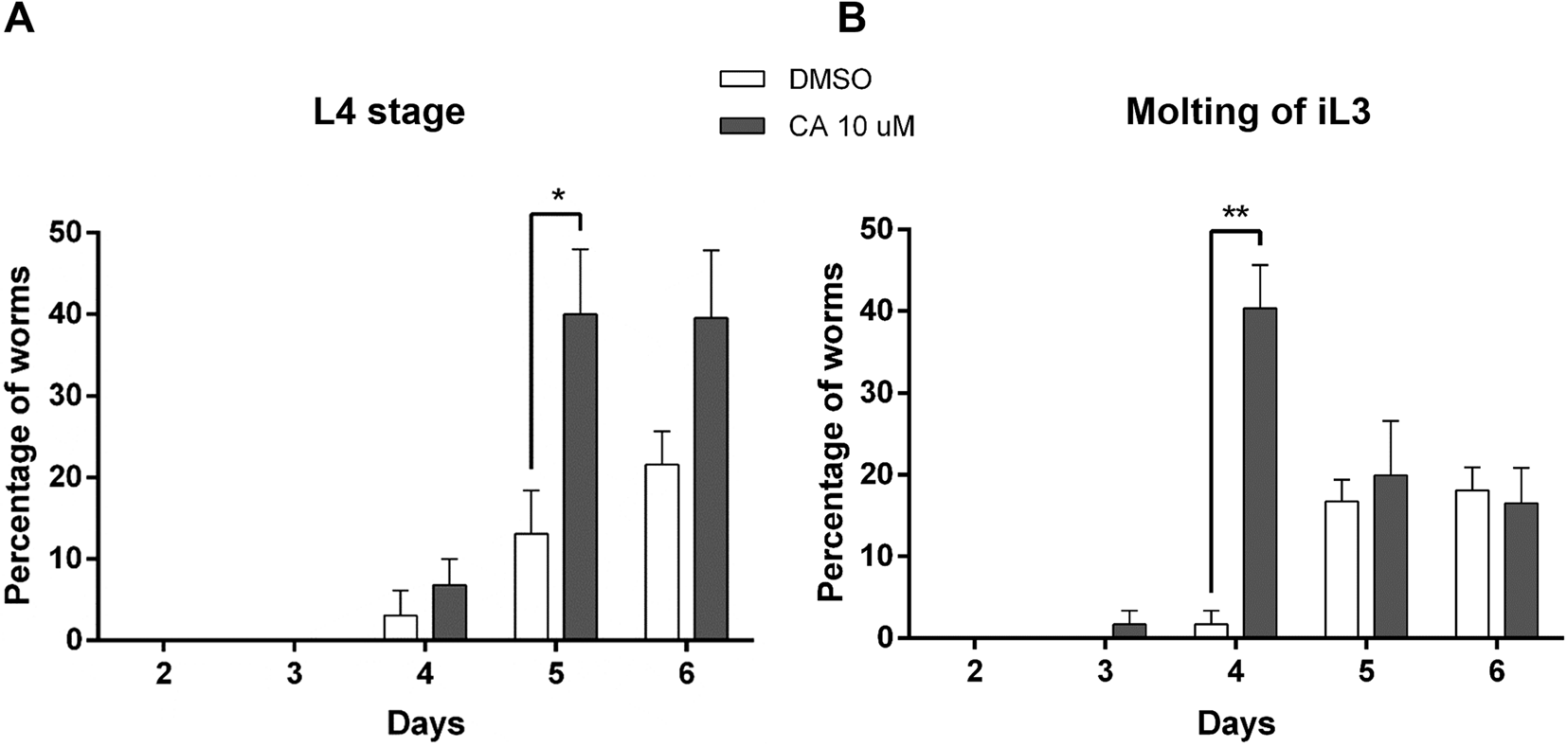
Cholestenoic acid accelerates the complete molting of infective third stage *D. immitis* larvae to L4. The iL3 stage larvae were collected from mosquitos and treated with cholestenoic acid (CA) or DMSO (**A**). The developmental changes were observed daily under a microscope. The iL3 that completed the molting from iL3 to L4 (**A**) were scored as well as the iL3 that were still undergoing molting (**B**). The number of free cuticles (left panel) found in the wells corresponded to larvae that had completed the molt and were L4. The figure shows a representative experiment performed in quadruplate using Prism 6.0 (Graph Pad Software, Inc.). The statistical significance was evaluated with an unpaired *t* test (**p < 0.005; *p < 0.05).

We then tested the effects of CA on the development of iL3 and found that the molting of iL3 started at day 3 when treated with CA, which was one day earlier than when iL3 were treated with DMSO (Fig. 8A). At day 4, the number of iL3 undergoing molting as well as the number of L4 were significantly higher with CA than DMSO (Fig. 8B).

## Discussion

The developing stages from the iL3 and L4 larval stages of *D. immitis* are critically important for preventing clinical disease, as the target stages for chemoprophylaxis of heartworm disease by MLs. Nevertheless, the signaling pathways governing the molting of iL3 larvae inside the dog are poorly understood in *D. immitis;* but it is an essential process for the parasite to develop and maintain its life cycle. In this research, we have identified for the first time an ortholog of DAF-12 from a filarial parasite as a possible novel target to interrupt the parasite development and eliminate the heartworm parasite *D. immitis*. Interestingly, DimDAF-12 shares some close structural homologies with the previously described NR of *C. elegans* CelDAF-12 and DAF-12s from Clade IV and V parasitic nematodes which have bacteria-feeding free-living stages (more than 30% identity in the whole sequence)^8,20,21^. Particularly, the typical NR sequences are conserved in the DBD (more than 94%) and the ligand-binding domain (LBD) (more than 40% identity) (Fig. 1). Based on the crystal structures released for AceDAF-12 and SstDAF-12 (respectively, 3up0 and 3gyt), we proposed a model for the 3D structure of DimDAF-12_LBD and predicted the binding mode of different ligands (Figs. 3, 4). DAs are the natural ligands of CelDAF-12 and have been shown to bind and activate SstDAF-12, AceDAF-12, AcaDAF-12, *N. americanus* DAF-12 and *Haemonchus contortus* DAF-12^10,18,20,21^. We showed by combining docking calculations and in vitro cell-based transactivation assays that the two forms of DAs (Δ4-DA and Δ7-DA) also bind strongly to DimDAF-12 (Figs. 2, 3, 4, 5). Nevertheless, DAs bind to and activate differently DAF-12 according to nematode species. Interestingly, the two isomers of DAs bound with a markedly higher affinity (× 100) to DimDAF-12 from the filarial parasite *D. immitis* (Fig. 2), than to CelDAF-12 and the other Clade IV and V parasitic DAF-12s investigated so far (EC_50_ = 0.21–0.65 nM for DimDAF-12 versus EC_50_ = 16–81 nM and 25–294 nM respectively for CelDAF-12 and parasitic nematode DAF-12)^20,21^ suggesting than DimDAF-12 was much more sensitive to DAs than all the other nematode DAF-12. Regarding the binding of DAs on DimDAF-12, our models did not predict any interaction between the C3 ketone group of Δ4- or Δ7-DA and surrounding side chains of DimDAF-12_LBD, whereas in AceDAF-12 and SstDAF-12, a H-bond interaction was found involving glutamine residues Q571 or Q637, respectively^20,21^. The equivalent residue Q713 in DimDAF-12 model was nevertheless found not far from the DA ketone group; however, at too long a distance for favoring an H-bond, but indicating a favorable polar environment. Interestingly, among the two biological assemblies of the AceDAF-12/Δ7-DA complex resolved in 3up0, one structure (chain A) did not show the direct H-bond interaction of Q571 with the ketone group of Δ7-DA, but interacted through a molecule of water bridge^21^. Moreover, the residue R674 has shown to be important for Δ4-DA-dependent activity of DimDAF-12 but not the Δ7-DA-dependent activity. This residue has also been shown critical in the activity of AceDAF-12 and CelDAF-12 but not SstDAF-12^21^. It is also interesting that there is evidence of species specificity in the steroid biosynthetic pathways. In *C. elegans*, cytochrome P450 DAF-9 is a limiting enzyme for DA biosynthesis, for which a gene homolog has yet to be identified in the genomes of the parasitic nematode *S. stercoralis* and *A. ceylanicum*^13,20,21^. Our analysis of the *D. immitis* genome also failed to identify a *daf-9* homolog, suggesting that the natural ligand of DimDAF-12 in *D. immitis* might not be a DA*.* This could be in agreement with the inability of nematode parasites to synthesize their own ligands including DAs, as previously suggested^4,20,21^ and their need to exploit host nutrients as triggers of their development. However, an orthologue of *daf-9* has been recently identified in *H. contortus* with a role in the synthesis of Δ7-DA and larval development^18^ as previously described for *C. elegans*^43^.

In contrast to threadworms and hookworms, *D. immitis* is an obligate parasite that is transmitted by the mosquito to the dog, which are two distinct, non-bacterial environments that might provide different ligands to regulate DimDAF-12 effects. DAF-9 has a similar function to mammalian CYP27A1, which synthesizes bile acid metabolites from cholesterol. Since *D. immitis* is auxotrophic for cholesterol^44^, we speculate that the vertebrate host provides the DimDAF-12 ligand based on previous observations in other parasitic nematodes that lack a pathway for DA synthesis^4,20,21^. Indeed, the mammalian bile acid metabolite 3β-hydroxy-5-cholestenoic acid (CA) has previously been shown to weakly activate CelDAF-12 and rescue the dauer formation of *daf-9* mutants^45^. Moreover, (25S)-diastereoisomer of CA has also been shown to stimulate the activity of AceDAF-12 and have a higher sensitivity to the receptor than DAs^21^. The fact that the CA binds to the DimDAF-12 LBP, with a high affinity (Fig. 7B), and activates the receptor of *D. immitis* at a concentration for optimal binding (EC_50_ = 178.7 nM, Fig. 7C) in the same range as that measured in circulating mammalian plasma (about 456 nM), suggests that CA of mammalian origin could be a natural ligand of DimDAF-12 (Fig. 7). This hypothesis is supported by our observation that CA also improves significantly the molting of iL3 into L4 (Fig. 8).

Since the ortholog of DAF-12 in *D. immitis* binds DAs with high affinity, this suggests the existence of a conserved DAF-12 downstream pathway^15,46^. Interestingly, the analysis of the *D. immitis* genome revealed that genes known to be regulated by CelDAF-12 are also conserved in *D. immitis*. Among them, we identified an homolog of the serine threonine kinase *lit-1*, that might play an important role in the development or in amphid channel morphogenesis of *D. immitis*, as shown in *C. elegans*^41^. Furthermore, CelDAF-12 has been shown to regulate larval development by activating the transcription of *let-7* family miRNAs (*mir-84* and *mir-241*) in *C. elegans*^14,15^. The family of *let-7* miRNAs has been identified in *Brugia pahangi*, the causative agent of zoonotic filariasis, and the upregulation of several *let-7* genes seems to correlate with the development of iL3 following host infection^47^. In the genome of *D. immitis*^39^, *let-7* miRNA family is also conserved suggesting that DimDAF-12 could possibly regulate larval development in a similar way as in *C. elegans*^4^.

Consistently, we showed that the Δ7-DA and Δ4-DA isoforms (Fig. 6) accelerates the molting of *D. immitis* from iL3 to L4 stage indicating that DimDAF-12 activation may play a key role in the development of this parasite. It is noteworthy that molting of iL3 to L4 in *D. immitis* is only possible in media supplemented with serum, suggesting that there might be a molecule in the serum that triggers the molting process by activating DimDAF-12^48,49^. This is strengthened by our data showing that CA binds and activates DimDAF-12 LBP (Fig. 7) and contributes to the molting of iL3 into L4 (Fig. 8), suggesting that the host mammalian environment may be the trigger of this process. Indeed, since helminths, including *D. immitis*, are unable to synthesize cholesterol, they rely on host cholesterol for critical biological processes that could include their development and growth inside the host^44^.

## Methods

### Amplification and cloning of DimDAF-12

Total RNA from male or female *D. immitis* worms and L3 larvae was isolated using the Trizol method (Invitrogen). cDNA was obtained by reverse transcription using Superscript III first strand (Invitrogen). The full length DimDAF-12 (nDi.2.2.2.t09652) was then amplified by High Fidelity Platinum Taq DNA polymerase (Invitrogen) using DimDAF-12Fw/DimDAF-12Rv primers (Table S4) encompassing the predicted initiation and stop codon of the respective ORF based on the *D. immitis* genome reference nDi.2.2.2 (https://www.nematodes.org/genomes/dirofilaria_immitis/). The product was subcloned into a pCR4 vector (Invitrogen) and the exact sequence was then confirmed by sequencing (McGill University/Genome Quebec Innovation Centre). Two DimDAF-12 cDNA sequence encoding two different isoforms of DimDAF-12 have been deposited in the GenBank database and are available under the respective accession number MN449986 (long form, DimDAF-12a and short form, MK820661).

### Constructs and cell-based transactivation assays

To construct Gal4:DimDAF-12_LBD, the LBD of DimDAF-12 (DimDAF-12_LBD; residues 587–828) was PCR amplified with Platinum Taq DNA polymerase High Fidelity (Invitrogen) using DimDAF-12-LBD-SmaIFw and DimDAF-12-SmaIRv primers (Table S4), which were flanked by SmaI restriction sites. The PCR product was then cloned after digestion with SmaI (New England BioLabs) into a mammalian expression vector, CMX (gift from D. J. Mangelsdorf)^10^, downstream of the DNA-binding domain of the Gal4 yeast transcription factor (Gal4-DBD).

NIH3T3 cells (ATCC) were cultured in DMEM (Dulbecco’s modified Eagle’s medium) with L-glutamine supplemented with 10% (v/v) FBS containing 100 U/ml penicillin and 100 μg/ml streptomycin.

DimDAF-12_T637V, DimDAF-12_R674K/M, DimDAF-12_Q713E/L and DimDAF-12_R640C mutants were created by respectively modifying the residue threonine at position 637 to a valine, the arginine at position 674 to a lysine or methionine, the glutamine 713 to a glutamate or leucine and the residue arginine at the position 640 to a cysteine using QuickChange XL Site-Directed Mutagenesis (Agilent Technologies) and primers including the desired mutation (Table S4). Double mutants were obtained using the same strategy and the corresponding primers. The presence of a mutation was then verified by sequencing.

To perform the cell-based transactivation assays, firefly luciferase under the control of the Gal4 response element (UAS, upstream activation sequence) was used as a reporter construct. 5 × 10^3^ NIH3T3 cells were seeded in 96-well plates and transiently transfected the following day in serum-free DMEM with 50 ng of DAF-12 receptors and 100 ng of reporter constructs using 2 µl of SuperFect Transfection Reagent (Qiagen). 10 ng of an additional reporter construct, Renilla luciferase was also co-transfected into cells and was used for normalization. *C. elegans* DAF-12 (CelDAF-12) (gift from D. J. Mangelsdorf)^10^ and empty CMX vector were used respectively as positive and negative controls. At 3 h after transfection, serum-free medium was replaced by complete medium and ligands or vehicle control was added. After 24 h incubation, the cells were lysed and luciferase and renilla activities were successively measured using the Dual-Glo luciferase assay system (Promega). The ligands used were: Δ4-dafachronic acid (Δ4-DA) (Cayman Chemical) and (25S)-Δ7-dafachronic acid (Δ7-DA) (Santa Cruz Biotechnology), and 3β-hydroxy-5-cholestenoic acid (CA) (Santa Cruz Biotechnology), a mammalian bile acid-like metabolite that shares a core structure with DAs (Figs. 4B, C and 7). The ligand stock solutions were dissolved in DMSO and the final concentration of DMSO was maintained at 0.1% in each well.

In order to draw a dose response curve, we first calculated the ratio of luminescence from the luciferase reporter to luminescence from the renilla reporter for each condition and replicate. The mean of this ratio was then calculated from triplicate or quadruplate assays and then the ratio of DimDAF-12- or CelDAF-12-transfected cells was normalized to empty vector-transfected cells that were treated under the same conditions. The curve was fitted using GraphPad Prism 6 using a sigmoidal, 4PL, standard curve type (four-parameter logistic curve). Data presented for the cell-based transactivation assays are representative experiments (shown as mean data ± SE from triplicate or quadruplicate replicates from one representative assay).

### Sequence alignment, homology model construction and validation

In the absence of a crystal structure, the 3D tertiary structure of *D. immitis* DAF-12_LBD was built by comparative modeling using Modeller 9.18 software^50,51^ and protocol previously described in David et al.^52^. Suitable template structures upon which to model the DimDAF-12 tertiary structure were retrieved using BLAST-P (Basic Local Alignment Search Tool) program and the protein data bank (pdb) database. Blast searches predicted that the crystal structures of *A. ceylanicum* (AceDAF-12; PDB ID: 3up0, resolved at 1.60 Å)^20^ and *S. stercoralis* DAF-12 (SstDAF-12; 3gyt, resolved at 2.40 Å)^21^ were suitable templates with highest homology to DimDAF-12 (BLAST-bit score: 282, 274 respectively) and excellent residue conservation (55 and 53% sequence identity, respectively). The ligands Δ4- and Δ7-DAs initially bound to 3up0 and 3gyt were not included in the homology model reconstruction of LBD. A pairwise sequence alignment between both 3up0 and 3gyt sequences was generated by Modeller script *salign* and then improved using a knowledge based approach. A multiple sequence alignment between DimDAF-12 and the pairwise alignment of crystal structures was then achieved using *align2D* script and used as input for Modeller 9.18. From this alignment, 100 structural models were generated by satisfaction of spatial restraints calculated by Modeller, which were ranked based on their DOPE (Discrete Optimized Potential Energy) score and their molecular pdf value of the objective function of Modeller^53^. We selected two homology models, one with the best DOPE score and another one with the best objective function value, and assessed their quality using various online scoring function servers; in particular: QMEAN (Qualitative Model Energy ANalysis) scoring function^54^ as a statistical potential, ProSA-web (Protein Structure Analysis) for evaluating energy profile and assessing the overall quality of the structure in term of Z score^55,56^, and PROCHECK for Ramachandran plots and listing the amino acids found in allowed and disallowed configurational regions (Table S2)^57^. The model with maximum number of residues in allowed regions in the Ramachandran plot was selected for further studies.

### Molecular docking studies

The in silico calculations for Δ4-DA, Δ7-DA and CA on the DimDAF-12 homology model were performed using the Autodock 4.2 program as previously described^52,58^. All ligand structures were prepared using Marvin Suite (https://www.chemaxon.com/products/marvin/marvinsketch/), from which 10 conformers were generated under the MMFF94 force field with correct stereochemistry, protonation states, and ring conformations. After 3D-alignment of conformers and calculation of RMSD under PyMOL for clustering, three representative conformers were selected for further docking calculations with Autodock 4.2. This program predicts top ranking poses with best scores and consists in two main programs, AutoGrid, which computes a three-dimensional grid of interaction energy based on macromolecular coordinates and Autodock, which performs the docking search.

The grid built by Autogrid 4 included the whole LBD of the DimDAF-12 receptor with 126, 126, and 98 points in x, y and z directions, with a grid spacing of 0.375 Å, to allow a complete search in the LBD structure for binding of DAs. For each ligand representative conformer, 100 runs were performed using the Lamarckian genetic algorithm. All the other parameters were kept as default. The generated poses were scored based on an estimated free energy of ligand binding calculated by Autodock, which can be considered as a binding affinity score. They were then clustered according to the similarity of their positions and conformations, with RMSD set at 2.0 Å, and finally ranked by their binding score (in each cluster). For each lowest energy pose of selected clusters, the number and nature of interacting residues were analyzed within the complex DimDAF-12/DA. Among these, particular interest was given to residues involved in the ligand binding previously described in the crystals 3up0 and 3gyt^20,21,42^.

### Development of *D. immitis*

*D. immitis* iL3 larvae (2005 Missouri strain) were obtained from FR3 Molecular Resources through BEI resources^59^. The iL3 were collected from infected mosquitoes as previously described^44^ and shipped overnight to McGill University. After several washes in PBS, the iL3 were incubated in RPMI-1640 with l-glutamine (Gibco) supplemented with 10% FBS, 0.1 mg/ml streptomycin, 100 U/ml penicillin, 0.04 mg/ml gentamycin, and 0.25 µg/ml amphotericin B. The next day, the iL3 were immediately transferred to a 24-well plate with 10–15 larvae per well and the medium replaced by fresh complete RPMI-1640 containing 10 µM of Δ7-DA or Δ4-DA or CA. Media was renewed every 2 days with fresh Δ7-DA. Animals were incubated at 37 °C and 5% CO_2_ and were observed every 24 h for 9 days under a stereomicroscope for any appearance of sheath or free cuticle as a signature of the molting process. Molting from the iL3 to the L4 larvae was recorded by scoring the number of larvae which had initiated but not completed the molt and the number of free cuticles into the medium. The percentage of iL3 that did not complete the molt and the percentage of L4 were calculated as the mean of iL3 or L4 from a quadruplate assay/total number of larvae × 100.

### Statistical analysis

All experiments were performed at least twice with triplicate or quadruplicate replicates with consistent results between experiments. Data presented for the cell-based transactivation assays are representative experiments (shown as mean data ± SE from triplicate or quadruplicate replicates from one representative assay). The effects of DimDAF-12 mutants in cell-based transactivation assays were analyzed by two-way ANOVA with multiple comparisons without correction using Prism 6.0 (Graph Pad Software, Inc.). The significance of small molecule effects on iL3 molting were analyzed using Student’s t test in Prism 6.0.

## Supplementary information

Supplementary information.

## Data Availability

Accession codes: Genbank accession number MN449986 for DimDAF-12a and MK820661 for DimDAF-12b.
