# Supplementary material for: The development of the dog heartworm is highly sensitive to sterols which activate the orthologue of the nuclear receptor DAF-12
Source: Sci Rep. 2020 Jul 8;10:11207. doi: 10.1038/s41598-020-67466-9 (PMC7343802; doi:10.1038/s41598-020-67466-9)
Supplement: Supplementary file 1 — Supplementary information. [file 41598_2020_67466_MOESM1_ESM.docx]

**The development of the dog heartworm is highly sensitive to sterols which activate the orthologue of the nuclear receptor DAF-12**

Thavy Long^1,2,*^, Mélanie Alberich^1^, François André^3^, Cécile Menez^1^, Roger K Prichard^2^, Anne Lespine^1,*^

^1^ INTHERES, Université de Toulouse, INRAE, ENVT, Toulouse Cedex 3, 31027, France

^2^  Institute of Parasitology, McGill University, Sainte-Anne-De-Bellevue, H9X3V9 QC, Canada

^3^ Université Paris-Saclay, CEA, CNRS, Institute for Integrative Biology of the Cell (I2BC), 91198, Gif-sur-Yvette, France

* Corresponding author: [thavy.long@mcgill.ca](mailto:thavy.long@mcgill.ca) (TL); [anne.lespine@inrae.fr](mailto:anne.lespine@inrae.fr) (AL)

**Supporting information**

**S1 Table.** Comparison of key residues inside the ligand binding pocket (LBP) between *Dirofilaria immitis*, *Ancylostoma ceylanicum* and *Strongyloides stercoralis* DAF-12 receptors. The LBP contains mainly non-polar residues that probably interact with the hydrophobic steroid core of DAs and few polar residues that maintain electrostatic interactions with the ligand oxygen atoms. T495/T562, respectively in Ace/SstDAF-12 are involved in H-bonding with carboxylate group of DAs; R532/R599, respectively in Ace/SstDAF-12 are involved in salt bridge with carboxylate group of DAs; T546 in Ace/SstDAF-12 are involved in H-bonding with DA carboxylate group and finally Q571/Q637, respectively in Ace/SstDAF-12 establish hydrogen bond with DAs C3 ketone group.

**S2 Table.** Comparison of scores for structure assessment of the models generated by Modeller with the best DOPE score (DimDAF-12_94) and the best molpdf score (DimDAF-12_46), with scores of crystal structures of AceDAF-12 (3up0) and SstDAF-12 (3gyt). Overall model quality was assessed by the scoring functions QMEAN of SwissModel (Qualitative Model Energy Analysis, (<https://swissmodel.expasy.org/qmean>), Z-score of ProSA-web (<https://prosa.services.came.sbg.ac.at/prosa.php>), and by the Ramachandran plot (PROCHECK, VADAR web site).

**S3 Table. Summary of binding energy observed between DimDAF-12_LBD and dafachronic acids or its structural analogs using Autodock.**

**S4 Table.** List of primer sequences used to amplify and clone *Dirofilaria immitis* DAF-12 cDNA sequence.

**S1 Fig. Sequence alignment of LBD from DimDAF12 and crystals of AceDAF-12 (3up0) and SstDAF-12 (3gyt).** The letters “C” below the sequence correspond to cysteines in AceDAF-12 that were mutated to serine to facilitate crystallization. The hash tags (#) correspond to various lysines in the native AceDAF-12 that were mutated into the SstDAF-12 corresponding residues at the same position [21]. The multiple alignment was performed with Clustal Omega using MView format for the color coding. The amino acids were shaded with colors based on their identity.

**S1 Table**

| DimDAF-12 | AceDAF-12_3up0 | SstDAF-12_3gyt | Validation by mutagenesis |
| --- | --- | --- | --- |
| Asn608 | Asn466 | Asn533 |  |
| Leu620 | Phe478 | Leu545 |  |
| Ile630 | Ile488 | Ile555 |  |
| Ile633 | Ile491 | Val558 |  |
| Ile634 | Met492 | Met559 |  |
| Ile636 | Val494 | Ile561 |  |
| Thr637 | Thr495 | Thr562 |  |
| Arg640 | Arg498 | Arg565 | R→C [20] |
| Val668 | Ile526 | Ile593 |  |
| Leu671 | Leu529 | Leu596 |  |
| Thr672 | Thr530 | Thr597 |  |
| Arg674 | Arg532 | Arg599 | R→K; R→M [20] |
| Gly675 | Gly533 | Gly600 |  |
| Arg678 | Arg536 | Val603 | R→V or V→R [20] |
| Trp686 | Trp544 | Trp611 | W→R [20] |
| Thr688 | Thr546 | Thr613 |  |
| Pro689 | Pro547 | Pro614 |  |
| Val690 | Thr548 | Val615 |  |
| Ile697 | Val555 | Ile621 |  |
| Met701 | Met559 | Met625 |  |
| Phe702 | Phe560 | Phe626 |  |
| Gln713 | Gln571 | Gln637 | Q→E [20] |
| Gly716 | Arg574 | Gly640 | R→K or G→R [21] |
| Phe717 | Phe575 | Phe641 |  |
| Ile800 | Ile657 | Val724 |  |
| Ser801 | Ser658 | Ala725 |  |
| Ala804 | Cys661 (mutated to Ser for crystallization) | Ala728 |  |
| Leu807 | Leu664 | Leu731 |  |
| Phe808 | Phe665 | Phe732 |  |
| Phe825 | Phe682 | Phe749 |  |

**S2 Table**

|  | DimDAF-12_94 | DimDAF-12_46 | AceDAF-12 | SstDAF-12 |
| --- | --- | --- | --- | --- |
| Modeller |  |  |  |  |
| DOPE score | -30978 | -30667 |  |  |
| Molpdf score | 6444.1 | 6437.9 |  |  |
| Qmean4 score (Z-score) | -1.75 | -2.01 | -0.29 | -2.32 |
| C_beta interaction energy | -1.69 | -1.40 | -0.09 | 0.96 |
| All-atom pairwise energy | -0.09 | -0.25 | 1.33 | 0.76 |
| Solvation energy | 0.85 | 0.74 | 1.89 | 2.05 |
| Torsion angle energy | -1.66 | -1.95 | -0.72 | -3.00 |
| Secondary structure agreement | -1.03 | -1.03 | -0.79 | 0.06 |
| Solvent accessibility agreement | 0.10 | 0.33 | -0.65 | -0.27 |
| ProSA-web |  |  |  |  |
| Overall model quality Z-score | -8.48 | -8.37 | -9.59 | -8.56 |
| VADAR Ramachadran plot (PROCHECK) (%) |  |  |  |  |
| Most favored | 97.3 | 96.8 | 97.6 | 92.2 |
| Allowed | 2.7 | 3.2 | 2.4 | 7.4 |
| Generously allowed | 0 | 0 | 0 | 0 |
| Disallowed | 0 | 0 | 0 | 0.4 |

**S3 Table**

| DimDAF-12 | Δ4-DA | Δ7-DA | CA |
| --- | --- | --- | --- |
| Docking characteristics  Number of poses | 19 | 50 | 22 |
| Binding energy (kcal/mol) | -13.3 | -13.5 | -13 |
| Hydrogen bonds | 2 | 2 | 2 |

Δ4-DA: Δ4-dafachronic acid

Δ7-DA: Δ7-dafachronic acid

CA: 3β-hydroxy-5-cholestenoic acid

**S4 Table**

| **Primer’s name** | **Sequence (5’-3’)** | **% GC** | **Tm** |
| --- | --- | --- | --- |
| DimDAF12Fw | ATGGCTGATATGAACAGTTTATTATC | 30.77 | 50.7 |
| DimDAF12Rv | TTAAGTAGTTTTGAAGAATTCTTTCGGA | 28.57 | 53.7 |
| CA_Fw | ACCTGCCAACCAAAGCGAGAAC | 54.55 | 62.4 |
| CA_Rv | TCAGGGTTATTGTCTCATGAGCG | 47.83 | 58.2 |
| cDNARv | GTTAAACATAGTTGGTGGTGTAC | 39.13 | 52.3 |
| Fw3 | CACATGCGAATCATGTAAAGC | 42.86 | 53.5 |
| Rv4bis | TTAAGTAGTTTTGAAGAATTCTTTCGGA | 28.57 | 47.3 |
| DimDAF12-LBD-SmaIFw | TTTTTCCCGGGTTATCAACTTAATTCAGCCGAA | 39.39 | 67.9 |
| DimDAF12-SmaIRv | AAAAACCCGGGTTTAAGTAGTTTTGAAGAATTCTT | 31.43 | 65.4 |
| GAL4Fw | ATGAAGCTACTGTCTTCTATC | 38.1 | 49.6 |
| pCMXRv | GCTTTAAATCTCTGTAGGTAGTTTG | 36 | 52.5 |
| DimDAF12T637VFw | CTTAATATCATAGATATCGTAATGCGAAGATTGGTTAA | 28.95 | 57.1 |
| DimDAF12T637VRv | TTAACCAATCTTCGCATTACGATATCTATGATATTAAG | 28.95 | 57.1 |
| DimDAF12R674KFw | gtagaaatgttgacgatgAAGggtgtgacgcgttttg | 45.95 | 66.6 |
| DimDAF12R674KRv | caaaacgcgtcacaccCTTcatcgtcaacatttctac | 45.95 | 66.6 |
| DimDAF12R678VFw | gatgcgtggtgtgacggtttttgatatggatcg | 48.48 | 66 |
| DimDAF12R678VRv | cgatccatatcaaaaaccgtcacaccacgcatc | 48.48 | 66 |
| DimDAF12Q713LFw | GGACTTCGTGATCGCCTAAAGGAAGGATTTATG | 45.45 | 63.8 |
| DimDAF12Q713LRv | CATAAATCCTTCCTTTAGGCGATCACGAAGTCC | 45.45 | 63.8 |
| DimDAF12R640CFw | CACAATGCGATGCTTGGTTAAAATG | 40 | 56.8 |
| DimDAF12R640CRv | ATATCTATGATATTAAGAATGTCAGTCGG | 31.03 | 52.8 |

**S1 Fig**

* * * ** ** *

DimDAF-12_LBD --YQLNSAELRALDIVRDAFACMNEPIEDSRKASYLKKATHNPTDILNIIDITMRRLVKM 644

3up0/AceDAF-12 GSYQLNAAELQALDLIQEAFKGMNDPMEQGRQATSFLKNEKSPADIMNIMDVTMRRFVKM 504

3gyt/SstDAF-12 GSYTLSEKDLKELDSIRDSFQCMNEPLDNDQQASTLAKKEHNPTDILNVMDITMRRLVKM 569

#

* ** ** * * *** * **

DimDAF-12_LBD AKKLPAFNDLSQDGKFALLKGGMVEMLTMRGVTRFDMDRKCWRTPVIPEESKISLEMFDQ 703

3up0/AceDAF-12 AKRLPAFNDLSQDGKFALLKGGMIEMLTVRGVRRFDSSSGSWTTPTLGESSEVSINMFDQ 563

3gyt/SstDAF-12 AKRLGAFNEISEAGKFSLLKGGMIEMLTIRGVTVFNADKGVWQTPVDG-HSQISFNMFDK 627

# # C

* **

DimDAF-12_LBD LKEGLRDRQKEGFMKFCESLHPDVRNNELAIDLIVLILLFDSNRDALLDPADRIIVVRHC 764

3up0/AceDAF-12 LNADVRSEQKMRFLQFFKIFHEDIRSNDLVISMIMLIVLF-SPRDSITDPEDRRIIARHH 623

3gyt/SstDAF-12 LRPDIKDTQKKGFLHFFNLLHSDVRKNDLAIDIIVLMVLFDSKREGLVSQQDKETVEKLH 688

C

** * **

DimDAF-12_LBD QEYQALLHRYMESMYGHEARLRYEHLPESLRILRTISQNAVTLFLGRVDPNQSEALPKEF 824

3up0/AceDAF-12 EQFSALLNRYLESLYGDDAHQLNEQLPTALRMLREISASSGMLFLGTVNTSEAEPLPREF 683

3gyt/SstDAF-12 RNYESLLHRYLYSIHKEEAEQRFASIPKALVALRKVAENAVTLFLGAGNTTEAASLPKEF 748

C # C

*

DimDAF-12_LBD FKTT- 828

3up0/AceDAF-12 FKVE- 687

3gyt/SstDAF-12 FATNY 753
